# Design, Synthesis and Biological Evaluation of Ligustrazine-Flavonoid Derivatives as Potential Anti-Tumor Agents

**DOI:** 10.3390/molecules23092187

**Published:** 2018-08-30

**Authors:** Hui Wang, Wenxi Zhang, Yatao Cheng, Xinyu Zhang, Nannan Xue, Gaorong Wu, Meng Chen, Kang Fang, Wenbo Guo, Fei Zhou, Herong Cui, Tao Ma, Penglong Wang, Haimin Lei

**Affiliations:** 1 School of Chinese Pharmacy, Beijing University of Chinese Medicine, Beijing 100102, China; 15652387323@163.com (H.W.); 20170931820@bucm.edu.cn (W.Z.); 20160931837@bucm.edu.cn (X.Z.); 18810612758@163.com (N.X.); gaorongwu09@163.com (G.W.); 18702267252@163.com (M.C.); 18364166994@163.com (K.F.); wb_guo@126.com (W.G.); zf116318@163.com (F.Z.); zhenzhu7696@163.com (H.C.); 2 International Cooperation Division, China Sinopharm International Corporation, Beijing 100102, China; chengyatao@sinopharm.com

**Keywords:** TMP-flavonoid derivatives, anti-tumor, human tumor cell lines, HUVEC-12, fluorescence staining, flow cytometry

## Abstract

In the clinic some anti-tumor drugs have shown damage to normal blood vessels, which could lead to vascular diseases. Therefore, it is necessary to evaluate the effects of anti-tumor drugs on normal blood vessels at the beginning of the drug design process. In this study, ligustrazine (TMP) and flavonoids were selected as raw materials. Sixteen novel TMP-flavonoid derivatives were designed and synthesized. Interestingly, compounds **14** and **16** were obtained by hydrolysis of a dihydroflavone to a chalcone under alkaline conditions. The cytotoxicity of the TMP-flavonoid derivatives was evaluated on five human tumor cell lines and one classical type of normal endothelial cell lines (HUVEC-12) by an MTT assay. Part of the derivatives showed better anti-tumor activities than the corresponding raw materials. Among them, compound **14** exhibited the closest activity to the positive control against the Bel-7402 cell line (IC_50_ = 10.74 ± 1.12 μM; DDP IC_50_ = 6.73 ± 0.37 μM) and had no toxicity on HUVEC-12 (IC_50_ > 40 μM). Subsequently, fluorescence staining and flow cytometry analysis indicated that compound **14** could induce apoptosis of Bel-7402 cell lines. Moreover, the structure-activity relationships of these derivatives were briefly discussed.

## 1. Introduction

As the world′s population ages and grows, the global burden of cancer continues to increase. Cancer is the leading cause of death and a major public health problem around the world [1,2]. Chemotherapy is commonly used for cancer treatment. In recent years, many chemotherapeutic drugs with high cytotoxicy have been discovered and used as first-line clinical drugs [3,4]. However, many anti-tumor drugs, such as cisplatin, paclitaxel and bleomycin [5,6,7], have serious side effects, including lethal hemoptysis and anemia [8,9]. The reasons are that the new tumor blood vessels were usually produced by an existing vascular system and considered to be the same as normal blood vessels. When drugs work, they have the same inhibition on normal endothelial cells (NECs) and tumor endothelial cells (TECs) [10,11,12]. Therefore, it is necessary to evaluate the effects of drugs on NECs while evaluating their anti-tumor activities. Compared with other drugs, Traditional Chinese Medicines (TCMs) have the advantages of lower toxicity and more targets. Therefore, it is possible to obtain high efficiency and low toxicity anti-tumor leading compounds by combining the anti-tumor ingredients from TCM.

In the clinic, TCMs with similar activities are often used together to realize synergistic effects. In the same way, it was possible to obtain more active compounds by synthesis of ingredients with similar effects [13,14]. TMP and flavonoids are two such ingredients [15]. Ligustrazine (TMP, 2,3,5,6-tetramethylpyrazine) is one of the active ingredients of *Ligusticum Chuanxiong Hort*, and it is widely used in occlusive cerebrovascular diseases. Previous studies have found that TMP and its derivatives could not only inhibit growth, invasion and metastasis of tumor cells, but also could reverse the multi-drug resistence and act as potential reversal drugs on P-gp [16,17,18,19]. It is widely used in the synthesis of leading compounds and a lot of compounds with significant effects have been obtained [20,21,22]. Flavonoids are a class of polyphenolic compounds widely distributed in the plant kingdom and abundantly consumed in the daily diet. They display potential therapeutic benefits in cancer treatment and may be considered as candidates for tumor therapeutic agents [23,24,25]. Flavonoids have been reported to be correlated to G1 and G2 cell cycle arrest and apoptosis promotion. Moreover, the flavonoids can reverse the multidrug resistance (MDR) of tumors and affect the expression and function of efflux transporters [26,27,28]. The nine flavonoids selected in this study are shown in Table 1. The main types of flavones were chosen, including flavones, flavonols, isoflavones and dihydroflavones.

In this study, we conjugated TMP and flavonoids, the main components of blood-activating drugs with anti-tumor activities, into the one molecule via ether bonds, constructing a number of new TMP-flavonoid derivatives. A variety of tumor cell lines were selected to evaluate the anti-tumor activities of these compounds, and human umbilical vein endothelial cells (HUVEC-12) as the NECs in vitro toxicity measurement model. 

## 2. Results

### 2.1. Chemical Synthesis

**Scheme 1:**
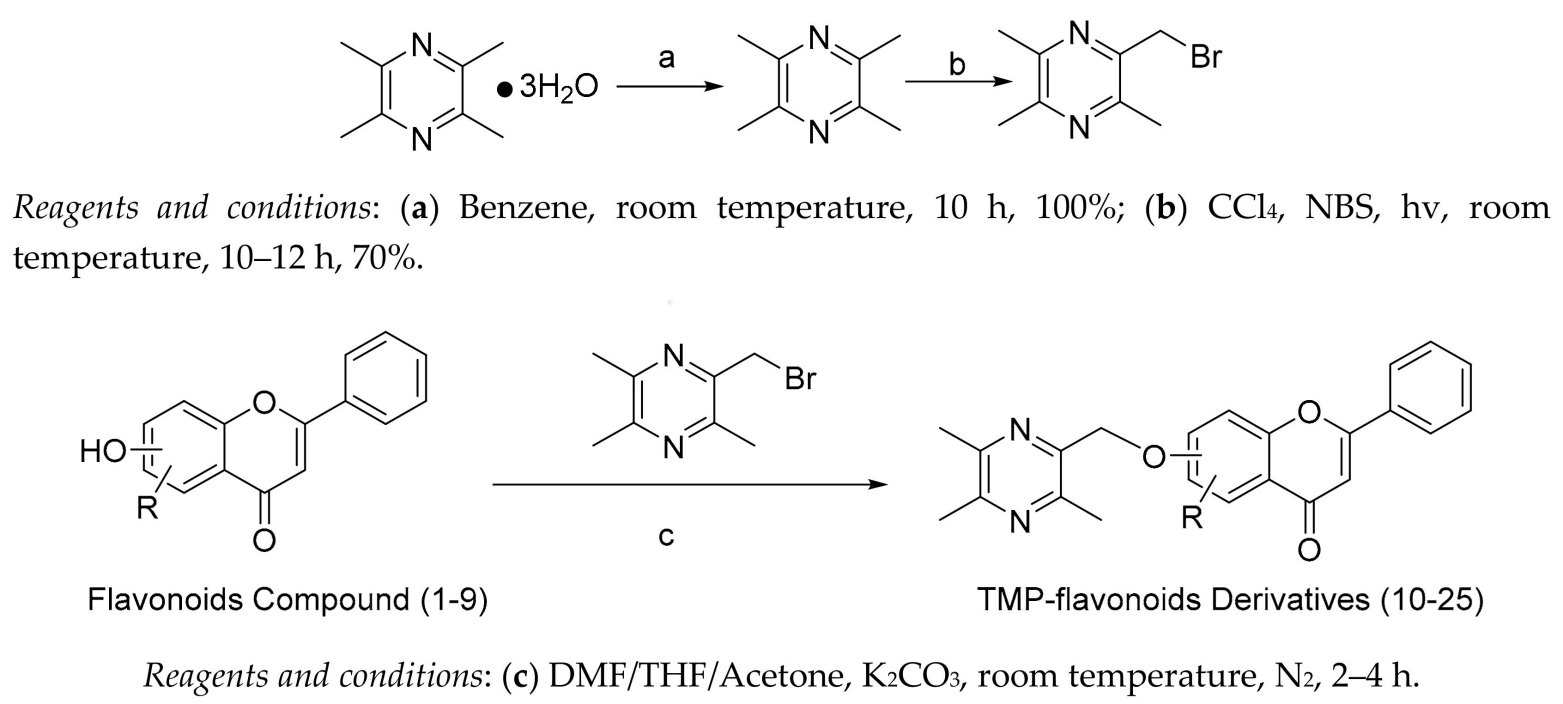
Synthesis routes to ligustrazine-flavonoid derivatives (**10**–**25**).

As shown in Scheme 1, we first synthesized the intermediate TMP-Br via a free radical reaction. Then, TMP-Br was linked to flavonoids via ether bonds to obtain TMP-flavonoid derivatives. Finally, end-products were obtained by crystallization. All TMP-flavonoid derivatives were synthesized by adopting a similar alkaline catalyst strength, which should be strictly controlled, as the flavanone structure is unstable under strong alkaline conditions. The C-O bond of C ring of naringenin derivatives was disconnected to form a chalcone structure (compounds **14**, **16**). Treatment of TMP-Br with daidzein in dry acetone at 75 °C for 2 h, afforded compound **10**. We had tried to synthesize more compounds, but the yield was too low. We could not obtain more compounds except these 16 derivatives (Table 2) and they are new compounds that had not been previously reported. All TMP-flavonoid derivatives were characterized by ^1^H-NMR, ^13^C-NMR and HRMS.

### 2.2. Biological Activities

#### 2.2.1. Cytotoxicity Assay Using Five Tumor Cell Lines

**Figure 1:**
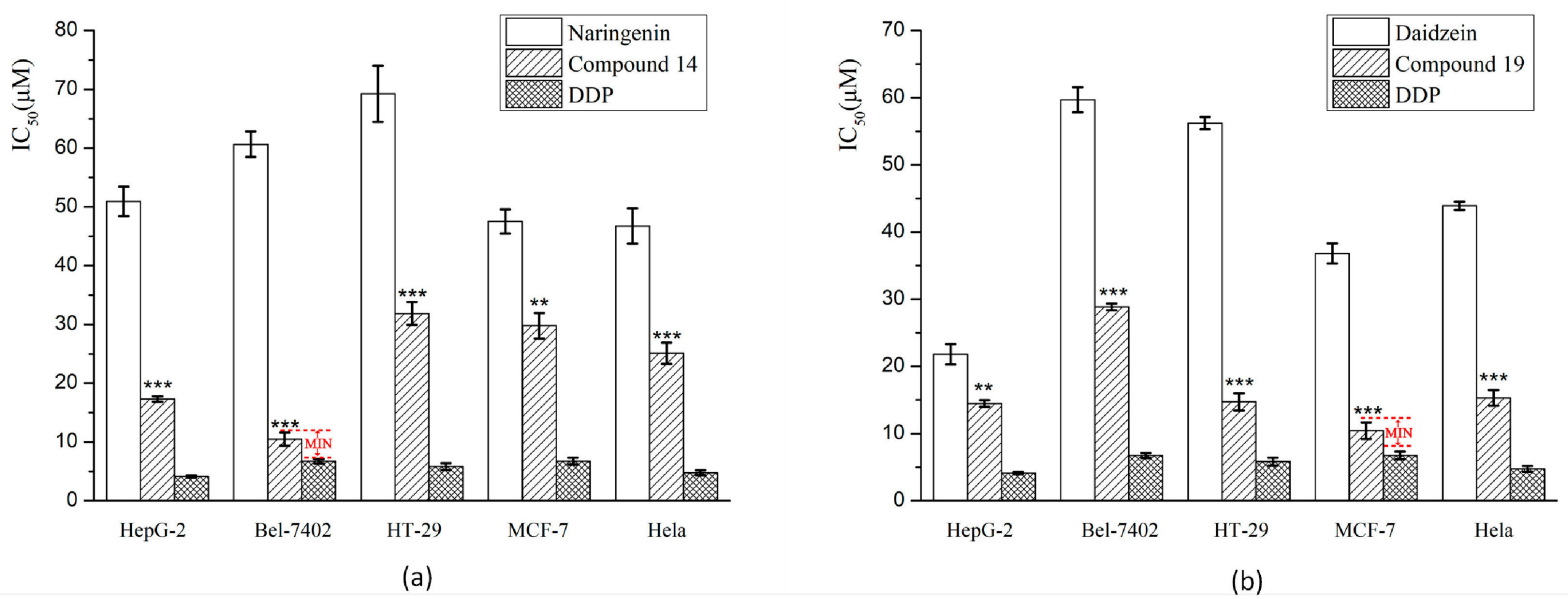
The IC_50_ values against HepG-2, Bel-7402, HT-29, MCF-7, HeLa. (**a**) Naringenin, compound **14**, DDP. (**b**) Daidzein, compound **19**, DDP. ** *p* < 0.001, *** *p* < 0.001, vs. Naringenin, Daidzein. The red mark indicated that the IC_50_ value of the compounds to the cells were closest to that of the DDP.

All the synthesized compounds were tested for their cytotoxicities on five tumor cell lines (HepG-2, Bel-7402, HT-29, MCF-7 and HeLa) using the standard MTT assay with DDP as positive control. The IC_50_ values of these compounds are summarized in Table 3. After combination, the activity of most synthesized compounds had not been improved significantly compared to the parent flavonoids. Among them, compounds **14** and **19** displayed lower IC_50_ values than the rest of compounds against Bel-7402, and MCF-7, respectively. As shown in Figure 1, the activity of compound **14** was significantly better than that of naringenin on all cell lines. Furthermore, compound **14** had the strongest inhibitory effect on Bel-7402 and slightly weaker than that of DDP. In the same way, the activity of compound **19** was superior to that of daidzein, and was shown to be slightly weaker than that of DDP against MCF-7.

In addition, it was observed that different types of flavonoids could lead to different cytotoxicities. The activity of derivatives with flavones, isoflavones and chalcone as mother nucleus was better than other derivatives, such as compound **14** (IC_50_ was 17.31 ± 0.47 μM, 10.74 ± 1.12 μM, 31.88 ± 1.96 μM, 29.79 ± 2.18 μM, 25.11 ± 1.80 μM aginst HepG-2, Bel-7402, HT-29, MCF-7 and HeLa respectively) and compound **19** (IC_50_ was 14.49 ± 0.48 μM, 28.87 ± 0.49 μM, 11.72 ± 1.29 μM, 10.43 ± 1.23 μM, 14.31 ± 1.17 μM aginst HepG-2, Bel-7402, HT-29, MCF-7 and HeLa respectively). Because of the limited number of derivatives, the relationships between the number and position of introduced TMP moieties and the activities of TMP-flavonoid derivatives need to be further explored.

#### 2.2.2. Cytotoxicity Assay Using HUVEC-12 Cell

In order to further investigate the effects of compounds with clear anti-tumor activities on NECs, MTT colorimetry was applied using human umbilical vein endothelial (HUVEC-12) cells. As shown in Table 4, when the concentration was 40 μM, compound **14** presented a little cytotoxicity. However, it showed a certain role in promoting cell proliferation and a concentration dependence at most concentrations. On the contrary, compound **19** appeared to have weak damaging effects and DDP had definite cytotoxic effects on cells (compounds **14** and **19** IC_50_ > 40 μM; DDP IC_50_ = 9.11 ± 0.54 μM) [29,30]. The results of compound **14** were consistent with expectations that the compounds had no effect on NECs. Therefore, compound **14** with the greater difference was selected for further mechanistic study.

#### 2.2.3. Morphological Analysis Using Giemsa and DAPI Staining

**Figure 2:**
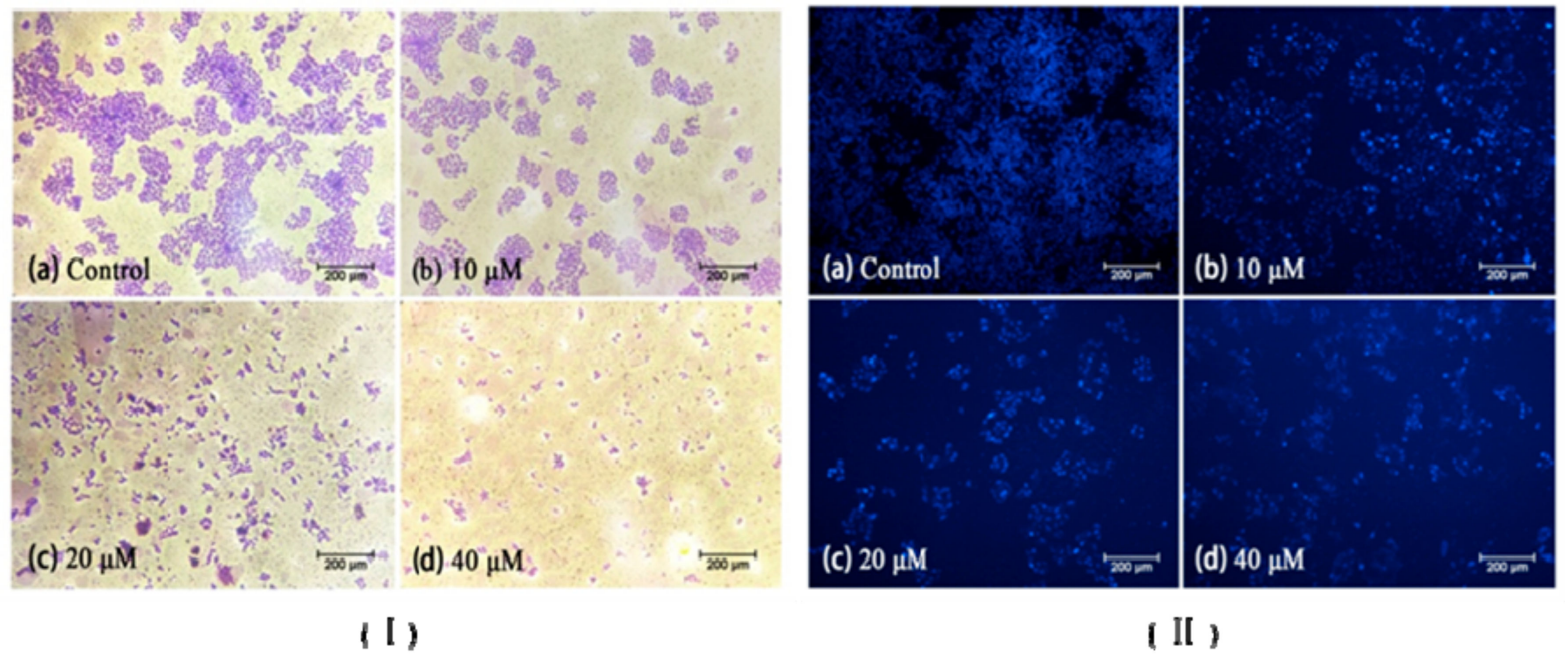
Gimesa (**I**) and DAPI (**II**) staining on Bel-7402 cells induced by compound **14** with different concentrations: (100×): (a) control group; (b) 10 μM; (c) 20 μM; (d) 40 μM. The cell morphology was observed under the fluorescence microscope. The most representative fields are shown.

To confirm the apoptopic morphological changes induced by compound **14**, Bel-7402 cells were treated with 0, 10, 20 and 40 μM of compound **14** for 72 h and then Giemsa staining and DAPI staining were performed. As shown in Figure 2I, the number and morphological changes of Bel-7402 cells significantly increased with increasing concentrations of compound **14**. When the dose reached 40 μM, there were mainly cell fragments rather than complete cells. In the same way, as we can see from Figure 2II, the cells treated with compound **14** showed morphological features of apoptosis, including a number decrease, nuclear fragmentation, cytoplasmic shrinkage, and the formation of apoptotic bodies with irregular shape while untreated cells displayed normal. 

#### 2.2.4. Apoptosis Analysis by Flow Cytometric Using Annexin V-FITC/Propidium Iodide (PI) Staining

**Figure 3:**
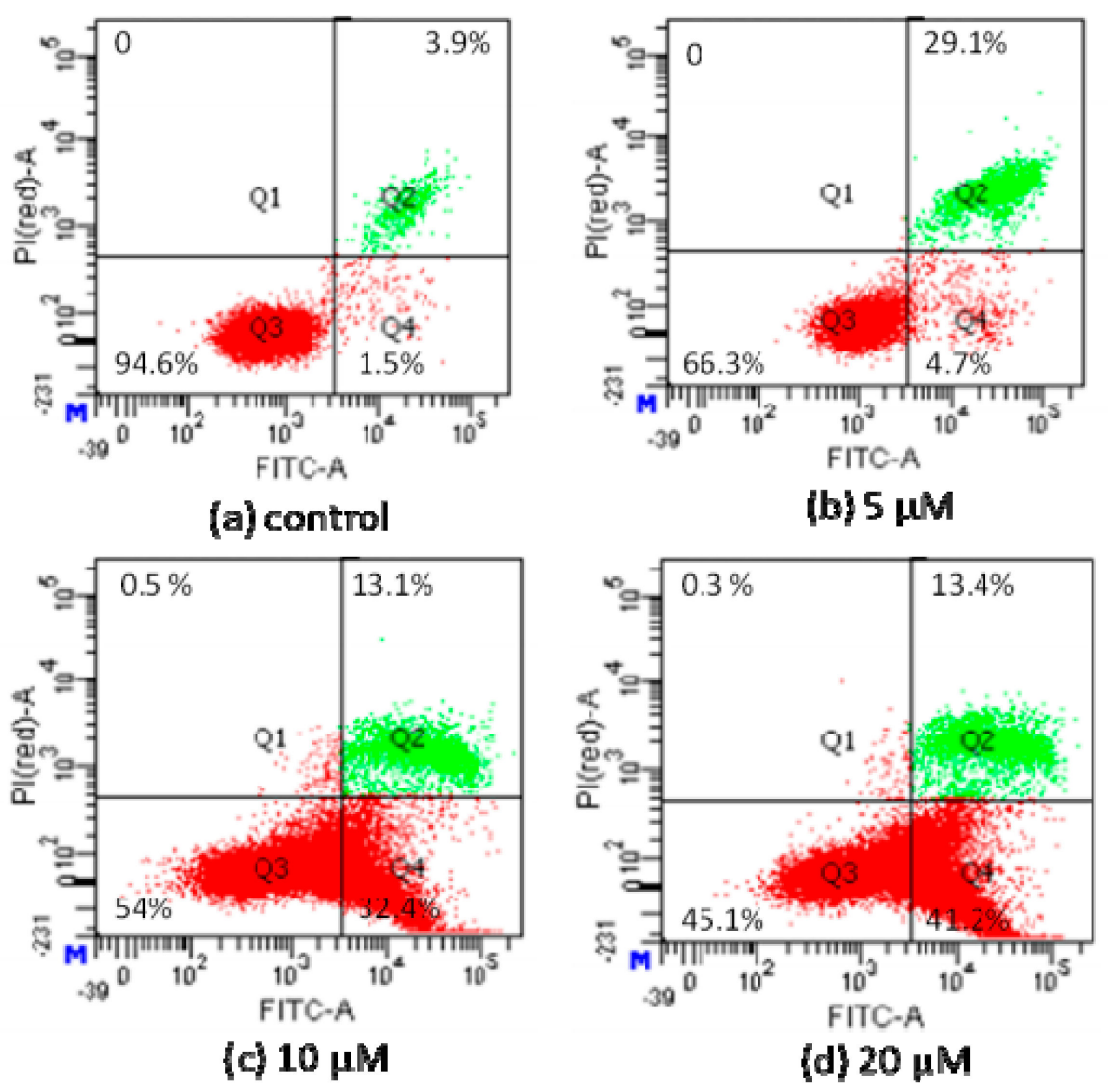
Apoptosis analysis of Bel-7402 cells induced by compound 14 using AnnexinV-FITC/PI staining: (**a**) control group; (**b**) 5 μM; (**c**) 10 μM; (**d**) 20 μM.

The effects of compound **14** on apoptosis of Bel-7402 cells were further determined by flow cytometric analysis. The cells were treated with compound **14** at four concentrations of 0, 5, 10, 20 μM and then stained with both annexin Annexin V-FITC and PI. The flow cytometry observed four quadrant images: necrotic (Q1; Annexin^−^/PI^+^), late apoptotic (Q2; Annexin^+^/PI^+^), intact (Q3; Annexin^−^/PI^−^), and early apoptotic (Q4; Annexin^+^/PI^−^) cells. The results are shown in Figure 3, where the apoptosis ratios (including the early and late apoptosis ratios) increased to 33.7% (5 μM), 45.5% (10 μM), 54.6% (20 μM) while that of control was 5.4%. Futhermore, the results indicated that compound **14** could induce Bel-7402 cells apoptosis in a concentration-dependent manner.

## 3. Discussion

In this study, although chalcone was not selected, compounds **14** and **16** with chalcone as the mother nucleus were obtained via synthesis. This was due to the fact the dihydroflavones hydrolyzed back into chalcone under alkaline conditions [31]. In addition, we analyzed the effects of the TMP-flavonoid derivatives on five tumor cell lines. The flavonoids selected showed clear anti-tumor activity, especially luteolin (IC_50_ was 11.83 ± 0.44 μM, 14.51 ± 0.68 μM, 19.24 ± 1.17 μM, 16.99 ± 0.31 μM, 21.28 ± 1.12 μM aginst HepG-2, Bel-7402, HT-29, MCF-7 and HeLa, respectively). The results confirmed the anti-tumor potential of luteolin in tumor therapy [32]. Most of the flavonoids had better inhibition on HepG-2 than other cell lines, which was consistent with previous studies [27,33]. After TMP was introduced, the selectivity of the derivatives changed. For example, compound **14** showed the strongest inhibitory effect on Bel-7402. Among all compounds, the compounds with isoflavones and chalcone as the mother nucleus had better anti-tumor activities than other compounds. Furthermore, the retention of 5, 7-OH could increase the inhibition of the compounds on tumor cells, such as compound **10** > **11**, compound **17** > **18**, compound **19** > **20**. That is to say, isoflavones, chalcone and its derivatives with 5, 7-OH substituents have great potential in tumor therapy [26]. Since vascular diseases caused by anti-tumor drugs are common, it is necessary to consider the effect of antitumor drugs on normal blood vessels at the beginning of the design plan. In this study, we considered not only the inhibition of drugs on tumor cell lines, but also the effects of drugs on normal vascular cells. The design idea of this study was from the perspective of clinical application. While compound **14** had a definite inhibition on Bel-7402, it also had a little proliferation on HUVEC-12. The selectivity was not detected for other samples, including positive ones. The results of staining and flow cytometric also demonstrated that compound **14** promoted the apoptosis of Bel-7402. The results are consistent with previous studies [26]. The flavonoids selected have been shown to be correlated to a G1 and G2 cell cycle arrest [27,28], so it is worth performing further studies on the effect of TMP-flavonoid derivatives in cell cycle determination. Based on the above, compound **14** showed bright prospects and should be further studied.

## 4. Materials and Methods

### 4.1. Materials and Instruments

Ligustrazine, luteolin, baicalein, apigenin, chrysin, quercetin, fisetin, genistein, daidzein, naringenin, and DDP were purchased from Aladdin Bio-Chem Technology Co., Ltd., (Shanghai, China) and Alfa Aesar Chemical Co., Ltd. (Tianjin, China). The purity of all the materials was more than 98%. All reagents were used without any further purification. Reagents of analytical reagent grade were provided by Beijing Chemical Plant (Beijing, China). ^1^H-NMR and ^13^C-NMR spectra were recorded on a DRX-500 spectrometer at 500/125 MHz (Bruker, Fällanden, Switzerland). ESI–MS were recorded on a Thermo Scientific TM LTQ Orbitrap XL hybrid FTMS instrument (Thermo Technologies, New York, NY, USA). Reactions were monitored by thin-layer chromatography (TLC) on precoated silica gel G-254 plates, which visualized in UV light (254 nm). Flash column chromatography was performed using 200–300 mesh silica gel (Qingdao Haiyang Chemical Co., Qingdao, China). Cellular morphologies were observed using an inverted fluorescence microscope (Olympus IX71, Tokyo, Japan). The yields were calculated based on the last step reaction.

Fetal bovine serum (FBS) and RPMI 1640 (DMEM) medium, Penicillin and streptomycin were obtained from Thermo Technologies. Gimesa, 6-diamidino-2-phenylindole (DAPI) were obtained from Molecular Probes/Invitrogen Life Technologies (Carlsbad, CA, USA). Five kinds of tumor cell lines were provided by the Institute of Peking Union Medical College: Hepatocellular cancer cell lines (HepG-2, Bel-7402), human breast cancer cell lines (MCF-7), human colon cancer cells (HT-29), human cervical cancer cells (HeLa), human umbilical vein endothelial cells (HUVEC-12).

### 4.2. Chemical Syntheses

*2-(Bromomethyl)-3,5,6-trimethylpyrazine (TMP-Br)*: TMP·3H_2_O (0.26 mol) was dissolved in benzene (120 mL) and refluxed for 10 h. After concentrating the solution, white solids (TMP) were obtained. TMP (0.15 mol) was dissolved in carbon tetrachloride (100 mL) and NBS (0.12 mol) was added in portions. The mixture was refluxed and illuminated by four 60 W tungsten light bulbs. After 12 h, the brown liquid was filtered and dried in vacuum. A brown crude oily product was obtained (TMP-Br), which was not purified further, as it caused a strong mucous membrane irritation. Yield: 70%.

*5-Hydroxy-2-phenyl-7-((3,5,6-trimethylpyrazin-2-yl)methoxy)-4H-chromen-4-one*, (**10**). TMP-Br (2.81 mmol) and chrysin (1.97 mmol) were dissolved in dry DMF (30 mL), then K_2_CO_3_ (10.87 mmol) was added in portions. The mixture was refluxed and kept at 75 °C for 2 h under a nitrogen atmosphere, the brown solution was filtered and concentrated under vacuum. The product was separated by flash chromatography with dichloromethane-acetone (10:1) as eluent and recrystallized from dichloromethane. White powder, m.p.: 156.2–157.1 °C, yield 58%. ^1^H-NMR (CDCl_3_) (ppm): *δ* 12.72 (s, 1H), 7.88–7.87 (m, 2H), 7.53–7.52 (m, 2H), 6.66 (s, 1H), 6.64 (s, 1H), 6.47 (s, 1H), 5.22 (s, 2H), 2.53 (s, 6H), 2.47 (s, 3H); ^13^C-NMR (CDCl_3_) (ppm) *δ* 182.61, 164.59, 164.18, 162.35, 157.83, 151.92, 150.09, 148.98, 144.71, 131.99, 131.42, 129.23, 126.42, 106.05, 99.07, 93.72, 70.47, 21.88, 21.55, 21.33, 20.74. MS (ESI) *m*/*z*: [M + H]^+^ 389.1510, calcd. for C_23_H_20_N_2_O_4_ 388.1423. 

*2-Phenyl-5,7-bis((3,5,6-trimethylpyrazin-2-yl)methoxy)-4H-chromen-4-one*, (**11**). TMP-Br (5.63 mmol) and chrysin (1.97 mmol) were dissolved in dry DMF (30 mL), then K_2_CO_3_ (10.87 mmol) was added in portions. The mixture was refluxed and kept at 75 °C for 2 h under a nitrogen atmosphere; the brown solution was filtered and concentrated under vacuum. The product was separated by flash chromatography with dichloromethane-acetone (10:1) as eluent and recrystallized from dichloromethane. White powder, m.p.: 223.1–224.2 °C, yield 60%. ^1^H-NMR (CDCl_3_) (ppm) *δ* 7.83–7.81 (m, 2H), 7.47–7.46 (m, 3H), 6.718 (d, *J* = 5.0 Hz, 2H), 6.58 (s, 1H), 5.30 (s, 2H), 5.20 (s, 2H), 2.69 (s, 3H), 2.57 (s, 3H), 2.51 (s, 6H), 2.49 (s, 3H), 2.47 (s, 3H); ^13^C-NMR (CDCl_3_) (ppm) δ 177.14, 162.73, 160.76, 159.75, 159.60, 151.94, 151.24, 150.88, 150.09, 149.02, 148.28, 145.37, 144.77, 131.72, 131.30, 129.07, 126.08, 110.09, 109.21, 98.65, 95.21, 71.30, 70.42, 21.87, 21.69, 21.57, 21.45, 20.82, 20.73. MS (ESI) *m*/*z*: [M + H]^+^ 523.2359, calcd. for C_31_H_30_N_4_O_4_ 522.2267.

*5-Hydroxy-2-phenyl-6,7-bis((3,5,6-trimethylpyrazin-2-yl)methoxy)-4H-chromen-4-one*, (**12**). TMP-Br (5.29 mmol) and baicalein (1.85 mmol) were dissolved in dry DMF (30 mL), then K_2_CO_3_ (10.87 mmol) was added in portions. The mixture was refluxed and kept at 75 °C for 2 h under a nitrogen atmosphere; the brown solution was filtered and concentrated under vacuum. The product was separated by flash chromatography with petroleum ether-acetone (8:1) as eluent and recrystallized from dichloromethane. Yellow powder, m.p.: 232.9–233.8 °C, yield 65%. ^1^H-NMR (CDCl_3_) (ppm) *δ* 12.67 (s, 1H), 7.93–7.83 (m, 2H), 7.62–7.45 (m, 3H), 6.81 (s, 1H), 6.67 (s, 1H), 5.25 (s, 2H), 5.14 (s, 2H), 2.61 (s, 3H), 2.57 (s, 3H), 2.53 (s, 6H), 2.49 (s, 3H), 2.45 (s, 3H); ^13^C-NMR (CDCl_3_) (ppm) *δ* 182.83, 164.16, 158.21, 153.89, 153.54, 151.94, 150.87, 150.54, 150.34, 148.81, 148.56, 146.40, 144.56, 132.00, 131.66, 131.47, 129.27, 126.40, 106.70, 105.83, 92.34, 74.26, 71.03, 21.91, 21.73, 21.54, 21.46, 20.83, 20.50. MS (ESI) *m*/*z*: [M + H]^+^ 539.2303, calcd. for C_31_H_30_N_4_O_5_ 538.2216.

*2-Phenyl-5,6,7-tris((3,5,6-trimethylpyrazin-2-yl)methoxy)-4H-chromen-4-one*, (**13**). TMP-Br (7.93 mmol) and baicalein (1.85 mmol) were dissolved in dry DMF (30 mL), then K_2_CO_3_ (10.87 mmol) was added in portions. The mixture was refluxed and kept at 75 °C for 2 h under a nitrogen atmosphere; the brown solution was filtered and concentrated in vacuum. The product was separated by flash chromatography with petroleum ether-acetone (8:1) as eluent and recrystallized from dichloromethane. White powder, m.p.: 185.4–186.9 °C, yield 55%. ^1^H-NMR (CDCl_3_) (ppm) *δ* 7.92–7.85 (m, 2H), 7.58–7.47 (m, 3H), 7.12 (s, 1H), 6.67 (s, 1H), 5.26 (s, 2H), 5.24 (s, 2H), 5.02 (s, 2H), 2.66 (s, 3H), 2.56 (s, 3H), 2.53 (s, 6H), 2.46 (s, 6H), 2.40 (s, 6H), 2.29 (s, 3H); ^13^C-NMR (CDCl_3_) (ppm) *δ* 177.14, 161.32, 157.01, 154.77, 152.02, 151.79, 150.67, 150.24, 149.78, 148.88, 148.47, 146.52, 146.24, 144.40, 139.88, 131.73, 131.46, 129.16, 126.13, 113.69, 108.54, 98.39, 76.27, 75.13, 70.92, 21.92, 21.73, 21.53, 21.41, 20.77, 20.36. MS (ESI) *m*/*z*: [M + H]^+^ 673.3163, calcd. for C_39_H_40_N_6_O_5_ 672.3060.

*(E)-1-(2-Hydroxy-4,6-bis((3,5,6-trimethylpyrazin-2-yl)methoxy)phenyl)-3-(4-((3,5,6-trimethylpyrazin-2-yl)methoxy)phenyl)prop-2-en-1-one* (**14**). TMP-Br (7.88 mmol) and naringenin (1.84 mmol) were dissolved in dry DMF (30 mL), then K_2_CO_3_ (7.24 mmol) was added in portions. The mixture was refluxed and kept at 75 °C for 2 h under a nitrogen atmosphere; the brown solution was filtered and concentrated under vacuum. The product was separated by flash chromatography with dichloromethane-acetone (10:1) as eluent and recrystallized from dichloromethane. Yellow powder, m.p.: 128.1–129.1 °C, yield 70%. ^1^H-NMR (CDCl_3_) (ppm) δ 14.39 (s, 1H), 7.62 (s, 2H), 7.02 (d, *J* = 8.7 Hz, 2H), 6.83 (d, *J* = 8.7 Hz, 2H), 6.26 (dd, *J* = 13.0, 2.1 Hz, 2H), 5.20 (s, 2H), 5.17 (d, *J* = 1.6 Hz, 4H), 2.60 (s, 6H), 2.53 (s, 18H), 2.49 (s, 3H); ^13^C-NMR (CDCl_3_) (ppm) δ 192.83, 168.48, 164.93, 161.57, 160.26, 152.03, 151.80, 150.03, 149.26, 149.10, 145.46, 144.95, 144.75, 142.25, 129.96, 128.68, 125.56, 115.04, 106.73, 95.71, 92.57, 70.58, 70.12, 70.12, 22.01, 21.83, 21.80, 21.59, 20.71, 20.67. MS (ESI) *m*/*z*: [M + H]^+^ 675.3306, calcd. for C_39_H_42_N_6_O_5_ 674.3217.

*5,7-bis((3,5,6-Trimethylpyrazin-2-yl)methoxy)-2-(4-((3,5,6-trimethylpyrazin-2-yl)methoxy)phenyl)chroman-4-one* (**15**), TMP-Br (7.88 mmol) and naringenin (1.84 mmol) were dissolved in dry DMF (30 mL), then K_2_CO_3_ (14.49 mmol) was added in portions. The mixture was refluxed and kept at 75 °C for 2 h under a nitrogen atmosphere; the brown solution was filtered and concentrated under vacuum. The product was separated by flash chromatography with dichloromethane-acetone (10:1) as eluent and recrystallized from dichloromethane. White powder, m.p.: 176.4–177.6 °C, yield 52%. ^1^H-NMR (CDCl_3_) (ppm) *δ* 7.36 (d, *J* = 8.5 Hz, 2H), 7.04 (d, *J* = 8.6 Hz, 2H), 6.45 (d, *J* = 1.5 Hz, 1H), 6.24 (d, *J* = 1.7 Hz, 1H), 5.33 (dd, *J* = 13.0, 2.0 Hz, 1H), 5.23 (s, 2H), 5.16 (s, 2H), 5.12 (s, 2H), 2.99 (dd, *J* = 16.4, 13.4 Hz, 1H), 2.71 (dd, *J* = 16.5, 2.5 Hz, 1H), 2.66 (s, 3H), 2.58 (s, 3H), 2.55 (s, 3H), 2.53 (s, 3H), 2.52 (s, 12H), 2.49 (s, 3H); ^13^C-NMR (CDCl_3_) (ppm) *δ* 188.86, 164.88, 164.66, 161.01, 159.01, 151.85, 151.49, 151.35, 150.79, 150.14, 150.04, 148.98, 148.77, 148.29, 145.63, 145.29, 144.82, 131.43, 127.85, 115.19, 106.66, 95.50, 95.20, 79.06, 70.98, 70.21, 70.15, 45.65, 21.88, 21.83, 21.78, 21.56, 21.49, 20.83, 20.76, 20.72. MS (ESI) *m*/*z*: [M + H]^+^ 675.3302, calcd. for C_39_H_42_N_6_O_5_ 674.3217.

*(E)-3-(4-((3,5,6-Trimethylpyrazin-2-yl)methoxy)phenyl)-1-(2,4,6-tris((3,5,6-trimethylpyrazin-2-yl)-methoxy)phenyl)prop-2-en-1-one* (**16**), TMP-Br (10.51 mmol) and naringenin (1.84 mmol) were dissolved in dry DMF (30 mL), then K_2_CO_3_ (14.49 mmol) was added in portions. The mixture was refluxed and kept at 75 °C for 2 h under a nitrogen atmosphere; the brown solution was filtered and concentrated under vacuum. The product was separated by flash chromatography with dichloromethane-acetone (10:1) as eluent and recrystallized from dichloromethane. Yellow powder, m.p.: 61.6–62.3 °C, yield 58%. ^1^H-NMR (CDCl_3_)(ppm) δ 7.32 (d, *J* = 8.7 Hz, 2H), 7.13 (d, *J* = 16.0 Hz, 1H), 6.95 (d, *J* = 8.7 Hz, 2H), 6.69 (d, *J* = 16.0 Hz, 1H), 6.54 (s, 2H), 5.16 (s, 4H), 5.09 (s, 4H), 2.61 (s, 3H), 2.58 (s, 3H), 2.54 (s, 3H), 2.53 (s, 3H), 2.52 (s, 6H), 2.43 (s, 6H), 2.41 (s, 12H); ^13^C-NMR (CDCl_3_) (ppm) δ 194.08, 160.90, 160.46, 157.39, 151.71, 151.66, 151.32, 150.63, 150.20, 150.14, 148.85, 148.23, 145.35, 144.73, 130.10, 127.99, 127.40, 115.22, 112.90, 93.83, 70.97 (2C), 70.29, 70.10, 21.90, 21.89, 21.77, 21.62, 21.59, 21.45, 20.81, 20.79, 20.73. MS (ESI) *m*/*z*: [M + H]^+^ 809.4166, calcd. for C_47_H_52_N_8_O_5_ 808.4061.

*5-Hydroxy-7-((3,5,6-trimethylpyrazin-2-yl)methoxy)-3-(4-((3,5,6-trimethylpyrazin-2-yl)methoxy)-phenyl)-4H-chromen-4-one* (**17**). TMP-Br (5.29 mmol) and genistein (1.85 mmol) were dissolved in dry DMF (30 mL), then K_2_CO_3_ (10.87 mmol) was added in portions. The mixture was refluxed and kept at 75 °C for 2 h under a nitrogen atmosphere; the brown solution was filtered and concentrated under vacuum. The product was separated by flash chromatography with dichloromethane-acetone (10:1) as eluent and recrystallized from dichloromethane. White powder, m.p.: 134.6–135.4 °C, yield 67%, ^1^H-NMR (CDCl_3_) (ppm) *δ* 12.82 (s, 1H), 7.86 (s, 1H), 7.45 (d, *J* = 8.6 Hz, 2H), 7.08 (d, *J* = 8.6 Hz, 2H), 6.54 (d, *J* = 1.9 Hz, 1H), 6.47 (d, *J* = 1.9 Hz, 1H), 5.21 (s, 2H), 5.19 (s, 2H), 2.60 (s, 3H), 2.59 (s, 3H), 2.53 (s, 12H); ^13^C-NMR (CDCl_3_) (ppm) *δ* 180.82, 164.40, 162.75, 158.80, 157.86, 152.79, 151.75, 151.26, 149.91, 148.94, 148.76, 145.66, 144.62, 130.28, 130.14, 123.65, 123.43, 115.69, 115.02, 106.56, 99.00, 93.34, 70.32, 69.99, 40.15, 28.35, 21.71, 21.60, 21.43, 20.57. MS (ESI) *m*/*z*: [M + H]^+^ 539.2296, calcd. for C_31_H_30_N_4_O_5_ 538.2216.

*5,7-bis((3,5,6-Trimethylpyrazin-2-yl)methoxy)-3-(4-((3,5,6-trimethylpyrazin-2-yl)methoxy)phenyl)-4H-chromen-4-one* (**18**). TMP-Br (7.93 mmol) and genistein (1.85 mmol) were dissolved in dry DMF (30 mL), then K_2_CO_3_ (10.87 mmol) was added in portions. The mixture was refluxed and kept at 75 °C for 2 h under a nitrogen atmosphere; the brown solution was filtered and concentrated in vacuum. The product was separated by flash chromatography with dichloromethane-acetone (10:1) as eluent and recrystallized from dichloromethane. White powder, m.p.: 188.2–189.1 °C, yield 53%. ^1^H-NMR (CDCl_3_) (ppm) *δ* 7.74 (s, 1H), 7.43 (d, *J* = 8.7 Hz, 2H), 7.05 (d, *J* = 8.7 Hz, 2H), 6.77 (d, *J* = 2.1 Hz, 1H), 6.62 (d, *J* = 2.1 Hz, 1H), 5.31 (s, 2H), 5.23 (s, 2H), 5.18 (s, 2H), 2.67 (s, 3H), 2.61 (s, 4H), 2.60 (s, 3H), 2.56 (s, 7H), 2.54 (s, 6H), 2.51 (s, 3H), 2.50 (s, 3H); ^13^C-NMR (CDCl_3_) (ppm) *δ* 174.88, 162.41, 160.09, 159.65, 158.44, 151.82, 151.21, 150.89, 150.05, 149.97, 148.88, 148.60, 148.08, 145.73, 145.16, 144.63, 130.39, 126.00, 124.91, 115.42, 114.76, 110.50, 98.36, 94.72, 71.20, 70.28, 69.97, 21.76, 21.66, 21.62, 21.45, 21.42, 21.33, 20.85, 20.61. MS (ESI) *m*/*z*: [M + H]^+^ 673.3164, calcd. for C_39_H_40_N_6_O_5_ 672.3060.

*7-Hydroxy-3-(4-((3,5,6-trimethylpyrazin-2-yl)methoxy)phenyl)-4H-chromen-4-one* (**19**). TMP-Br (2.81 mmol) and daidzein (1.97 mmol) were dissolved in dry acetone (30 mL), then K_2_CO_3_ (10.87 mmol) was added in portions. The mixture was refluxed for 2 h under a nitrogen atmosphere; the brown solution was filtered and concentrated under vacuum. The product was separated by flash chromatography with petroleum ether-acetone (10:1) as eluent and recrystallized from dichloromethane. White powder, m.p.: 213.1–214.2 °C, yield 51%. ^1^H-NMR (DMSO-d_6_) (ppm) δ 9.52 (s, 1H), 8.33 (s, 1H), 7.98 (d, *J* = 8.9 Hz, 1H), 7.35 (d, *J* = 8.5 Hz, 2H), 7.09 (dd, *J* = 8.9, 2.3 Hz, 1H), 6.77 (d, *J* = 8.6 Hz, 2H), 5.27 (s, 2H), 2.46 (s, 3H), 2.40 (s, 6H); ^13^C-NMR (DMSO-d_6_) (ppm) *δ* 175.15, 163.08, 157.72, 153.67, 151.81, 149.86, 148.94, 145.00, 130.54, 127.48, 124.19, 122.80, 118.35, 115.44, 114.92, 102.06, 70.38, 21.76, 21.46, 20.62. MS (ESI) *m*/*z*: [M + H]^+^ 389.1506, calcd. for C_23_H_20_N_2_O_4_ 388.1423.

*7-((3,5,6-Trimethylpyrazin-2-yl)methoxy)-3-(4-((3,5,6-trimethylpyrazin-2-yl)methoxy)phenyl)-4H-chromen-4-one* (**20**). TMP-Br (5.63 mmol) and daidzein (1.97 mmol) were dissolved in dry DMF (30 mL), then K_2_CO_3_ (10.87 mmol) was added in portions. The mixture was refluxed and kept at 75 °C for 2 h under a nitrogen atmosphere; the brown solution was filtered and concentrated under vacuum. The product was separated by flash chromatography with petroleum ether-acetone (10:1) as eluent and recrystallized from dichloromethane. White solid, m.p.: 185.4–186.9 °C, yield 62%. ^1^H NMR (CDCl_3_) (ppm) *δ* 8.223 (d, *J* = 9 Hz, 1H), 7.94 (s, 1H), 7.51 (d, *J* = 8.5 Hz, 2H), 7.09 (dd, *J* = 8.9, 2.3 Hz, 1H), 6.77 (d, *J* = 8.6 Hz, 2H), 5.27 (s, 2H), 5.21 (s, 2H), 2.62 (s, 3H), 2.61 (s, 3H), 2.56 (s, 6H), 2.55 (s, 6H); ^13^C NMR (CDCl_3_) (ppm) *δ* 175.77, 162.80, 158.57, 157.77, 152.16, 151.83, 151.25, 149.98, 148.90, 148.68, 145.70, 144.61, 130.16, 127.91, 124.84, 124.71, 118.75, 114.93, 114.88, 101.45, 70.38, 69.98, 21.76, 21.66, 21.45, 21.44, 20.62. MS (ESI) *m*/*z*: [M + H]^+^ 523.2351, calcd. for C_31_H_30_N_4_O_4_ 522.2267.

*2-(3,4-Dihydroxyphenyl)-3,5,7-tris((3,5,6-trimethylpyrazin-2-yl)methoxy)-4H-chromen-4-one* (**21**). TMP-Br (7.09 mmol) and quercetin (1.66 mmol) were dissolved in dry DMF (30 mL), then K_2_CO_3_ (10.87 mmol) was added in portions. The mixture was refluxed and kept at 75 °C for 2 h under a nitrogen atmosphere; the brown solution was filtered and concentrated under vacuum. The product was separated by flash chromatography with dichloromethane-acetone (15:1) as eluent and recrystallized from dichloromethane. Yellow powder, m.p.: 121.2–122.3 °C, yield 65%. ^1^H-NMR (CDCl_3_) (ppm) *δ* 12.63 (s, 1H), 7.48 (s, 1H), 7.44 (d, *J* = 8.5 Hz, 1H), 7.05 (d, *J* = 8.5 Hz, 1H), 6.53 (d, *J* = 1.6 Hz, 1H), 6.45 (d, *J* = 1.5 Hz, 1H), 5.21 (s, 2H), 5.19 (s, 2H), 5.18 (s, 2H), 2.58 (s, 3H), 2.57 (s, 3H), 2.56 (s, 3H), 2.52 (s, 9H), 2.51 (s, 3H), 2.42 (s, 3H), 2.40 (s, 3H) ; ^13^C-NMR (CDCl_3_) (ppm) *δ* 178.92, 164.45, 162.18, 156.94, 156.85, 151.92, 151.87, 151.13, 150.03, 149.95, 149.49, 149.02, 148.82, 148.64, 148.35, 148.24, 145.45, 145.13, 144.73, 137.59, 126.04, 121.31, 117.19, 116.91, 106.50, 98.73, 93.23, 73.15, 72.00, 70.38, 21.85, 21.68, 21.55, 21.37, 21.06, 20.71, 20.59, 20.34. MS (ESI) *m*/*z*: [M + H]^+^ 705.3043, calcd. for C_39_H_40_N_6_O_7_ 704.2958.

*2-(3,4-bis((3,5,6-Trimethylpyrazin-2-yl)methoxy)phenyl)-3,5,7-tris((3,5,6-trimethylpyrazin-2-yl)-methoxy)-4H-chromen-4-one* (**22**). TMP-Br (11.83 mmol) and quercetin (1.66 mmol) were dissolved in dry DMF (30 mL), then K_2_CO_3_ (10.87 mmol) was added in portions. The mixture was refluxed and kept at 75 °C for 2 h under a nitrogen atmosphere; the brown solution was filtered and concentrated under vacuum. The product was separated by flash chromatography with dichloromethane-acetone (15:1) as eluent and recrystallized from dichloromethane. White powder, m.p.: 179.4–180.1 °C, yield 67%. ^1^H-NMR (CDCl_3_) (ppm) *δ* 7.75 (s, 1H), 7.63 (dd, *J* = 8.6, 1.7 Hz, 1H), 7.08 (d, *J* = 8.7 Hz, 1H), 6.74 (s, 1H), 6.70 (d, *J* = 1.8 Hz, 1H), 5.37 (s, 2H), 5.27 (s, 2H), 5.24 (s, 2H), 5.22 (s, 2H), 5.01 (s, 2H), 2.74 (s, 3H), 2.65 (s, 3H), 2.61 (s, 3H), 2.55 (s, 9H), 2.53 (s, 4H), 2.52 (s, 15H), 2.50 (s, 3H), 2.39 (s, 3H), 2.32 (s, 3H); ^13^C-NMR (CDCl_3_) (ppm) *δ* 173.50, 162.49, 159.57, 158.53, 153.02, 151.84, 151.31, 151.18, 151.12, 150.77, 150.29, 150.18, 150.07, 149.98, 148.88, 148.49, 148.11, 148.02, 145.98, 145.54, 145.35, 145.19, 144.61, 139.67, 123.86, 122.56, 114.44, 113.34, 110.02, 98.20, 94.63, 72.83, 71.24, 71.05, 70.91, 70.32, 21.78, 21.71, 21.68, 21.52, 21.47, 21.39, 21.37, 21.29, 20.86, 20.73, 20.63, 20.57. MS (ESI) *m*/*z*: [M + H]^+^ 973.4751, calcd. for C_55_H_60_N_10_O_7_ 972.4646.

*2-(3,4-bis((3,5,6-Trimethylpyrazin-2-yl)methoxy)phenyl)-5-hydroxy-7-((3,5,6-trimethylpyrazin-2-yl)-methoxy)-4H-chromen-4-one* (**23**). TMP-Br (7.49 mmol) and luteolin (1.75 mmol) were dissolved in dry DMF (30 mL), then K_2_CO_3_ (10.87 mmol) was added in portions. The mixture was refluxed and kept at 75 °C for 2 h under a nitrogen atmosphere; the brown solution was filtered and concentrated under vacuum. The product was separated by flash chromatography with dichloromethane-acetone (10:1) as eluent and recrystallized from dichloromethane. Yellow powder, m.p.: 163.8–164.7 °C, yield 55%. ^1^H-NMR (CDCl_3_) (ppm) *δ* 12.79 (s, 1H), 7.66 (d, *J* = 1.9 Hz, 1H), 7.49 (dd, *J* = 8.5, 2.0 Hz, 1H), 7.17 (d, *J* = 8.6 Hz, 1H), 6.63 (d, *J* = 2.1 Hz, 1H), 6.56 (s, 1H), 6.46 (d, *J* = 2.1 Hz, 1H), 5.28 (s, 2H), 5.26 (s, 2H), 5.22 (s, 2H), 2.59 (s, 3H), 2.57 (s, 3H), 2.57 (s, 3H), 2.53 (s, 9H), 2.51 (s, 3H), 2.51 (s, 3H), 2.50 (s, 3H); ^13^C-NMR (CDCl_3_) (ppm) δ 182.50, 164.45, 163.90, 162.35, 157.72, 151.97, 151.88, 151.63, 151.56, 150.36, 150.26, 150.08, 149.00, 148.74, 145.42, 145.18, 144.78, 124.32, 120.58, 114.00, 112.64, 106.02, 104.90, 98.98, 93.65, 71.36, 71.04, 70.48, 21.84, 21.56, 21.53, 20.74, 20.70. MS (ESI) *m*/*z*: [M + H]^+^ 689.3098, calcd. for C_39_H_40_N_6_O_6_ 688.3009.

*5,7-bis((3,5,6-Trimethylpyrazin-2-yl)methoxy)-2-(4-((3,5,6-trimethylpyrazin-2-yl)methoxy)phenyl)-4H-chromen-4-one* (**24**). TMP-Br (7.93 mmol) and apigenin (1.85 mmol) were dissolved in dry DMF (30 mL), then K_2_CO_3_ (10.87 mmol) was added in portions. The mixture was refluxed and kept at 75 °C for 2 h under a nitrogen atmosphere; the brown solution was filtered and concentrated under vacuum. The product was separated by flash chromatography with dichloromethane-acetone (8:1) as eluent and recrystallized from dichloromethane. White powder, m.p.: 180.9–181.5 °C, yield 55%. ^1^H-NMR (CDCl_3_) (ppm) *δ* 7.78 (d, *J* = 8.8 Hz, 2H), 7.10 (d, *J* = 8.8 Hz, 2H), 6.72 (d, *J* = 10.4 Hz, 2H), 6.51 (s, 1H), 5.31 (s, 2H), 5.22 (s, 4H), 2.71 (s, 3H), 2.59 (s, 3H), 2.59 (s, 3H), 2.53 (s, 6H), 2.52 (s, 6H), 2.51 (s, 3H), 2.49 (s, 3H); ^13^C-NMR (CDCl_3_) (ppm) *δ* 177.13, 162.59, 161.10, 160.65, 159.65, 159.55, 151.91, 151.67, 151.18, 150.86, 150.08, 149.00, 148.93, 148.27, 145.43, 145.25, 144.79, 127.73, 124.48, 115.36, 110.00, 107.94, 98.59, 95.18, 71.28, 70.39, 70.17, 21.86, 21.81, 21.67, 21.56, 21.44, 20.80, 20.72. MS (ESI) *m*/*z*: [M + H]^+^ 673.3158, calcd. for C_39_H_40_N_6_O_5_ 672.3060.

*2-(3,4-bis((3,5,6-Trimethylpyrazin-2-yl)methoxy)phenyl)-3,7-bis((3,5,6-trimethylpyrazin-2-yl)-methoxy)-4H-chromen-4-one* (**25**). TMP-Br (9.99 mmol) and fisetin (1.75 mmol) were dissolved in dry DMF (30 mL), then K_2_CO_3_ (10.87 mmol) was added in portions. The mixture was refluxed and kept at 75 °C for 2 h under a nitrogen atmosphere; the brown solution was filtered and concentrated under vacuum. The product was separated by flash chromatography with dichloromethane-acetone (10:1) as eluent and recrystallized from dichloromethane. White powder, m.p.: 162.7–163.1 °C, yield 48%. ^1^H-NMR (CDCl_3_) (ppm) *δ* 8.17 (d, *J* = 8.6 Hz, 1H), 7.81 (s, 1H), 7.69 (d, *J* = 8.6 Hz, 1H), 7.11 (s, 2H), 7.05 (s, 1H), 5.29 (s, 2H), 5.27 (s, 2H), 5.22 (s, 2H), 2.68 (s, 3H), 2.61 (s, 3H), 2.53 (s, 9H), 2.51 (s, 12H), 2.48 (s, 3H), 2.41 (s, 3H), 2.33 (s, 3H); ^13^C-NMR (CDCl_3_) (ppm) *δ* 174.55, 163.03, 156.86, 155.57, 151.97, 151.45, 151.25, 151.05, 150.75, 150.28, 150.18, 150.12, 149.03, 148.68, 148.24, 145.97, 145.70, 145.46, 144.74, 139.42, 127.34, 124.11, 123.02, 121.32, 118.52, 117.92, 116.91, 114.88, 114.82, 113.54, 101.45, 72.99, 71.27, 71.04, 70.54, 21.88, 21.81, 21.77, 21.64, 21.57, 21.51, 21.43, 20.74, 20.68. MS (ESI) *m*/*z*: [M + H]^+^ 823.3950, calcd. for C_47_H_50_N_8_O_6_ 822.3853.

### 4.3. Bio-Evaluation Methods

#### 4.3.1. Drugs

Ligustrazine, luteolin, baicalein, apigenin, chrysin, quercetin, fisetin, genistein, daidzein, naringenin, DDP and TMP-flavonoid derivatives (compounds **10**–**25**). All samples were dissolved in DMSO and prepared to 10^4^ μM. The final DMSO concentration did not exceed 0.1% (*V*/*V*). 

#### 4.3.2. Cell Culture

Five kinds of tumor cell lines and one normal endothelial cell were routinely cultured respectively in RPMI 1640 medium supplemented with 10% (*V*/*V*) FBS and 1% (*V*/*V*) penicillin-streptomycin, incubated at 37 °C in a 5% CO_2_ containing incubator.

#### 4.3.3. Cytotoxicity Assay Using Five Tumor Cell Lines

The five tumor cell lines were plated onto 96-well sterile plates in 100 μL/well of medium at a density of 3.5 × 10^3^ cells per well and incubated at 37 °C with 5% CO_2_ for 24 h. Then luteolin, baicalein, apigenin, chrysin, quercetin, fisetin, genistein, daidzein, naringenin, DDP and compounds **10**–**25** were added at various concentrations (5, 10, 20, 40, 50 μΜ). Each plate contained control group, blank group, drug group and positive group (DDP). After 72 h, 20 μL MTT in phosphate buffered saline (PBS, 5 mg/mL) was added to each well and the plates were incubated at 37 °C for 4 h, then removing the liquid and adding dimethyl sulfoxide (DMSO, 100 μL) to dissolve the MTT formazan. The optical density (OD) for each well was measured on a BIORAD 550 spectrophotometer plate reader (Bio-Gene Technology Ltd., Guangzhou, China) at a wavelength of 490 nm. All tests were carried out three times in parallel. The proliferation inhibition rates of tumor cells were calculated by {1 − [OD_490_ (Drug group) − OD_490_ (Blank group)]/[OD_490_ (Control group) − OD_490_ (Blank group)]} × 100%; the IC_50_ values were defined as the concentration of compounds that produced a 50% proliferation inhibition of surviving cells and calculated using the following equation: logIC_50_ = X_m_ − log2 × (∑P − 0.5), Where X_m_ = logC_max_, ∑P = sum of proliferation inhibition rates, the number 0.5 is an empirical constant. We first tested the activities of TMP-flavonoid derivatives with concentrations of 10 and 20 μM respectively. When compounds’ inhibition rate was not beyond 35% at a concentration of 20 μM, the compounds would not be considered to study further.

#### 4.3.4. Cytotoxicity Assay Using HUVEC-12 Cell

The HUVEC-12 cell were seeded in 96-well plates (6.0 × 10^3^ cells/ well), and incubated at 37 °C in a 5% CO_2_ containing incubator. After 24 h, the medium was replaced with fresh medium containing the compounds to be tested. Compounds **14**, **19** and DDP were added in final concentrations ranging from 2.5 to 40 μM. Each plate contained control group, blank group and drug group. After 24 h, 20 μL MTT was added to each well and the plates were incubated at 37 °C for 4 h, then removing the liquid and adding dimethyl sulfoxide (DMSO) (100 μL) to dissolve the MTT formazan. The optical density (OD) for each well was measured on a BIORAD 550 spectrophotometer plate reader at a wavelength of 490 nm. All tests were carried out three times in parallel. The proliferation inhibition rates of tumor cells were calculated by {1 − [OD_490_ (Drug group) − OD_490_ (Blank group)]/[OD_490_ (Control group) − OD_490_ (Blank group)]} × 100%.

#### 4.3.5. Morphological Analysis Using Giemsa and DAPI Staining

The Bel-7402 cells were plated onto 12-well sterile plates at a density of 2.4 × 10^4^ cells per well and incubated at 37 °C in a 5% CO_2_ containing incubator. Then the cells were incubated in the presence of compound **14** at various concentrations (0, 10, 20, 40 μΜ) for 72 h. Then cells were washed with PBS twice, fixed with cold ethanol and 4% paraformaldehyde (pH = 7.4) for 10 min respectively and washed with PBS again. Then fixed cells were stained with 6% Giemsa solution or DAPI at the concentration of 1 mg/mL for 5 min in the dark. Finally, the cells washed with water and dried. The cell morphological changes were observed by fluorescent microscopy and images were captured by digital camera [34,35].

#### 4.3.6. Apoptosis Analysis by Flow Cytometric Using Annexin V-FITC/Propidium Iodide (PI) Staining

The Bel-7402 cells were plated onto 6-well sterile plates (4.8 × 10^4^ cells/well) and placed at 37 °C with 5% CO_2_ for 24 h. Then compound **14** at various concentrations (0, 5, 10, 20 μΜ) was added. After 72 h, all cells were collected respectively with the right amount of trypsin (without EDTA) digestion. Then the cells were washed with cold PBS twice and centrifuged at 1000 rpm for 5 min. The harvested cells were resuspended in 200 μL binding buffer, which contained 10 μL Annexin V-FITC and PI. After avoided light reaction for 15 min, the cells were analyzed with a flow cytometer [36].

### 4.4. Statistical Analysis

All data were expressed as the means ± standard deviation (SD) of three replications. The statistical analysis was performed by SPSS software (Version 20.0, International Business Machines Corp. New York, NY, USA) to analyze the variance. One-way analysis of variance (ANOVA) followed by the least significant difference (LSD) post-hoc test for multiple comparisons. A *p*-value of less than 0.05 was considered significant.

## 5. Conclusions

Using TMP and flavonoids as starting materials, we have designed and synthesized sixteen novel TMP-flavnoid derivatives through conjugation of anti-tumor bioactive compounds via ether bonds. Among them, compounds **14** and **16** were obtained via dihydroflavone hydrolysis to chalcones under alkaline conditions. As the flavovoids and TMP showed broad anti-tumor activities, we chose five different human tumor cell lines to evaluate the TMP-flavonoid derivatives. In vitro chemosensitivity testing showed that most of compounds had certain anti-tumor activity against the HeLa, MCF-7, HT-29, HepG-2, Bel-7402 cell lines. Part of derivatives showed better anti-tumor activities than the raw materials. Besides, compounds **14** and **19** exhibited activities close to that of the positive control (DDP) against the Bel-7402 and MCF-7 cell lines, respectively (IC_50_ = 10.49 ± 1.12, 10.43 ± 1.23 μM; DDP IC_50_ = 6.73 ± 0.37, 6.75 ± 0.57 μM). Then, the effects of compounds **14** and **19** on NECs were determined using HUVEC-12 cells. Compound **14** showed pro-proliferation effects while compound **19** appeared to cause minor damage on cells and DDP had similar cytotoxic effects on HUVEC-12 and tumor cells (compounds **14** and **19** IC_50_ > 40 μM; DDP IC_50_ = 9.11 ± 0.54 μM). Subsequently, the results of fluorescence staining and flow cytometry analysis indicated that compound **14** could induce apoptosis in the Bel-7402 cell line. All results suggested that compound **14** has bright prospects. In addition, the discussion of structure-activity relationships indicated that isoflavones, chalcone and its derivatives with 5, 7-OH substituents have great potential on tumor therapy. Moreover, the attempt to apply structure combination to discover more efficient and multi-effective anti-tumor leading compounds from TCM formulations is viable.

## Figures and Tables

**Table 1:** The structures of the nine flavonoid compounds.

Classification	Flavonoids’ Structure	Flavonoid Compounds (1–9)	
**Flavone**	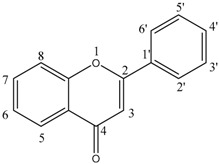	Luteolin (**1**)	5, 7, 3′, 4′—OH
		Baicalein (**2**)	5, 6, 7—OH
		Apigenin (**3**)	5, 7 ,3′—OH
		Chrysin (**4**)	5, 7—OH
**Flavonol**	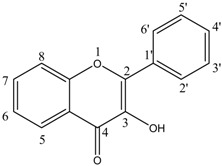	Quercetin (**5**)	5, 7, 3′, 4′—OH
		Fisetin (**6**)	7, 3′, 4′—OH
**Isoflavone**	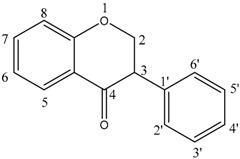	Genistein (**7**)	5, 7, 4′—OH
		Daidzein (**8**)	7, 4′—OH
**Flavanone**	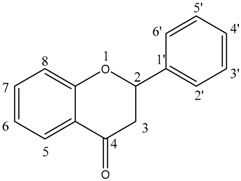	Naringenin (**9**)	5, 7, 4′—OH

**Table 2:** The structures of ligustrazine-flavonoid derivatives **10**–**25**.

No.	Structure	No.	Structure
**10**	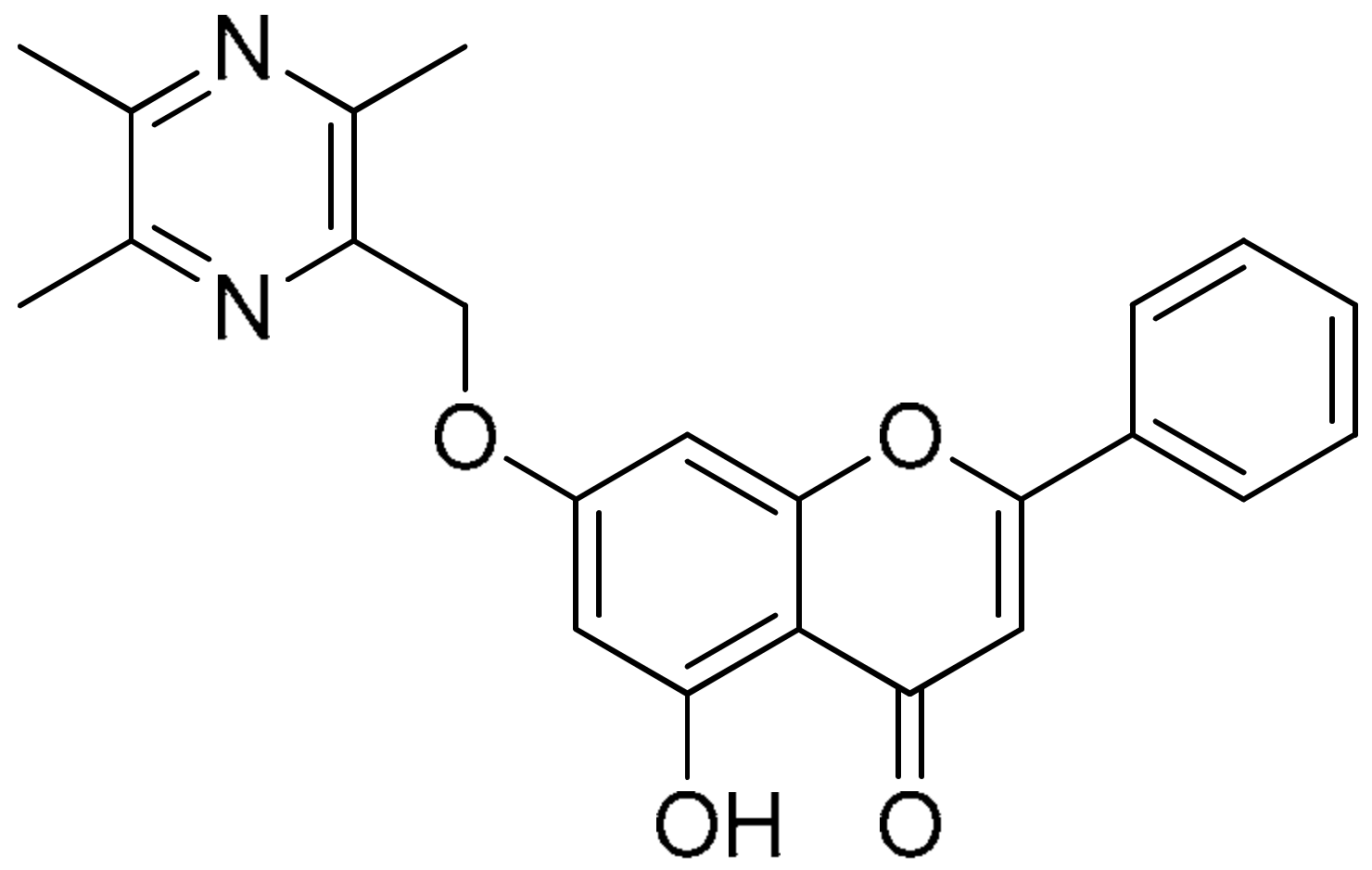	**11**	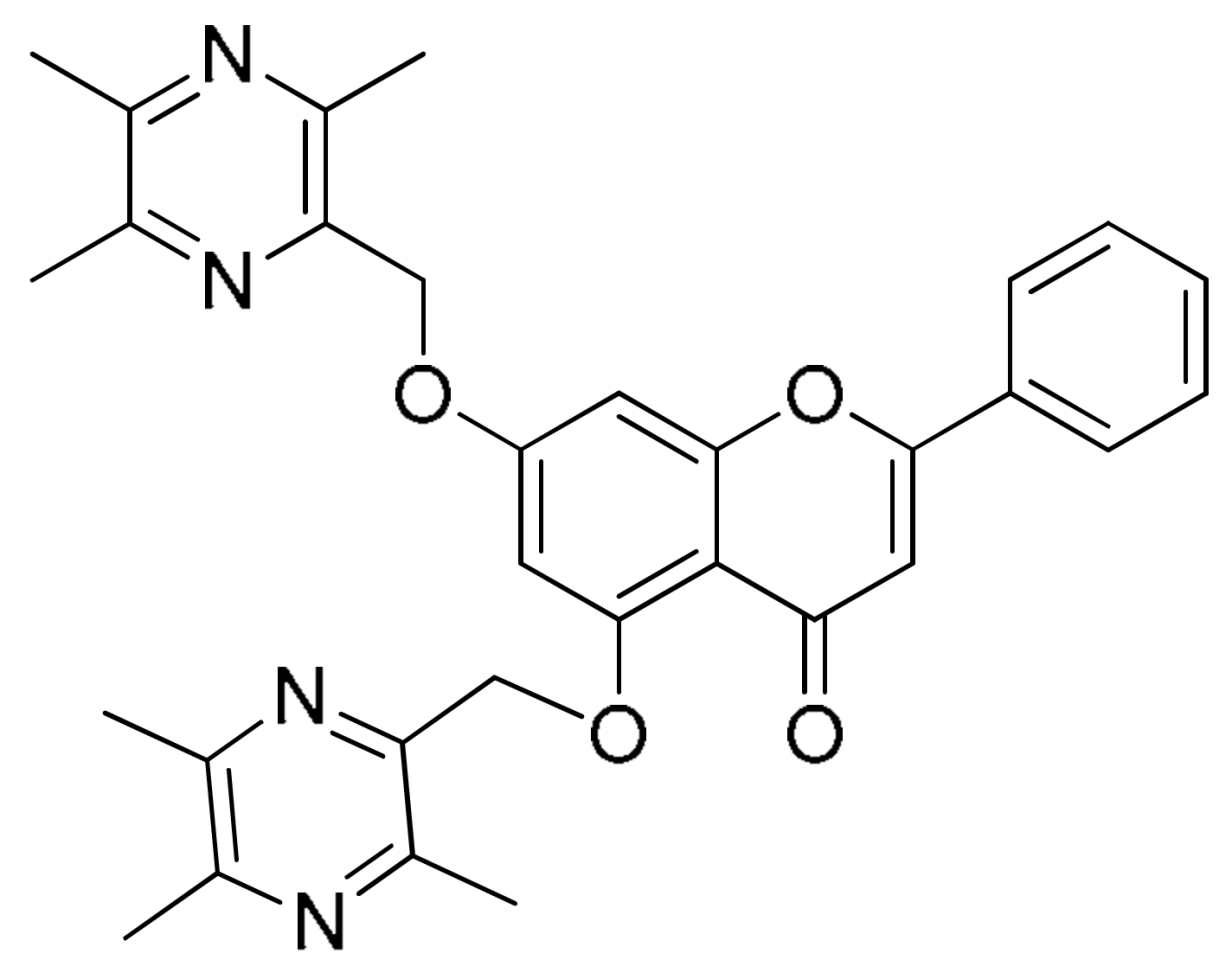
**12**	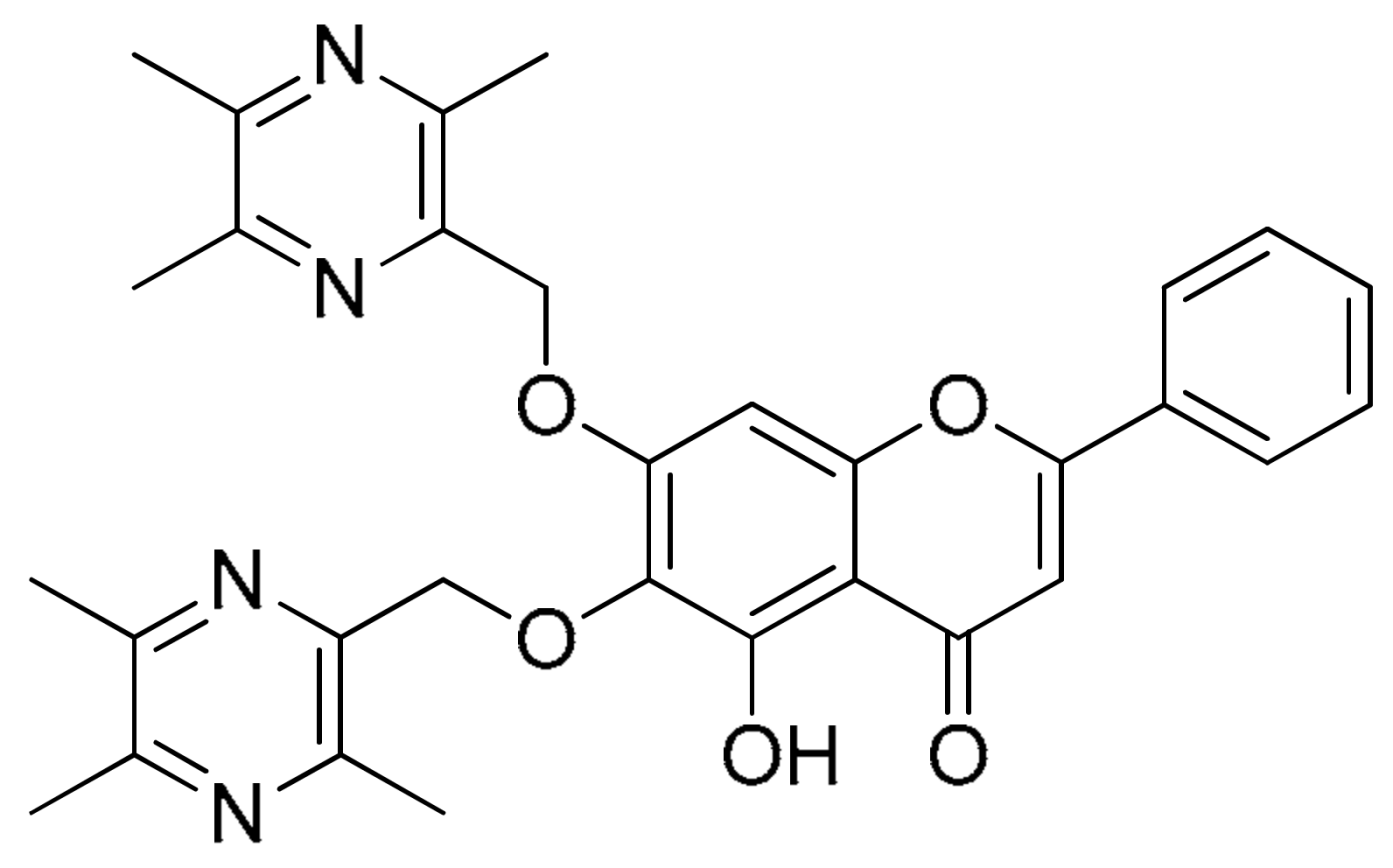	**13**	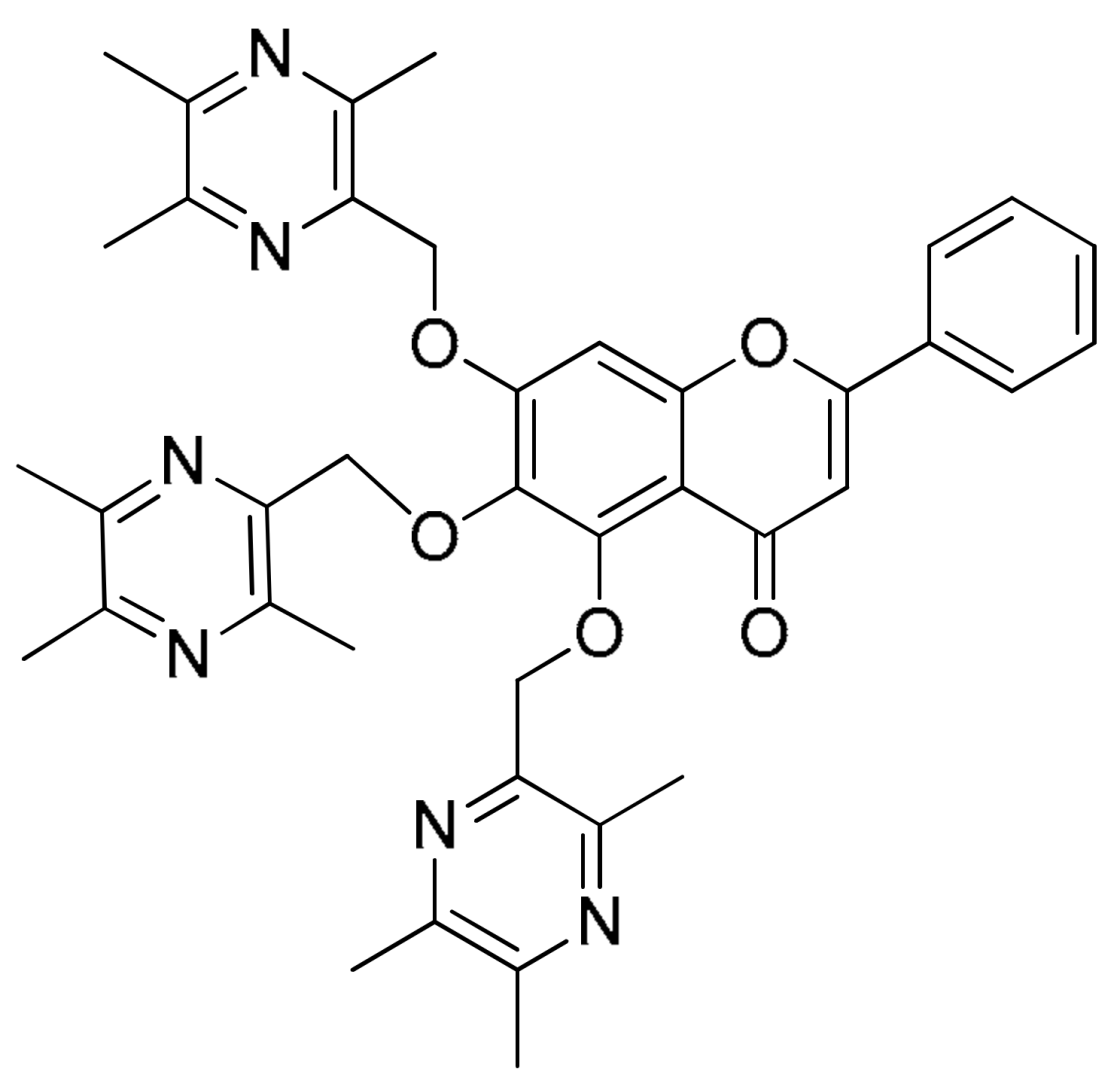
**14**	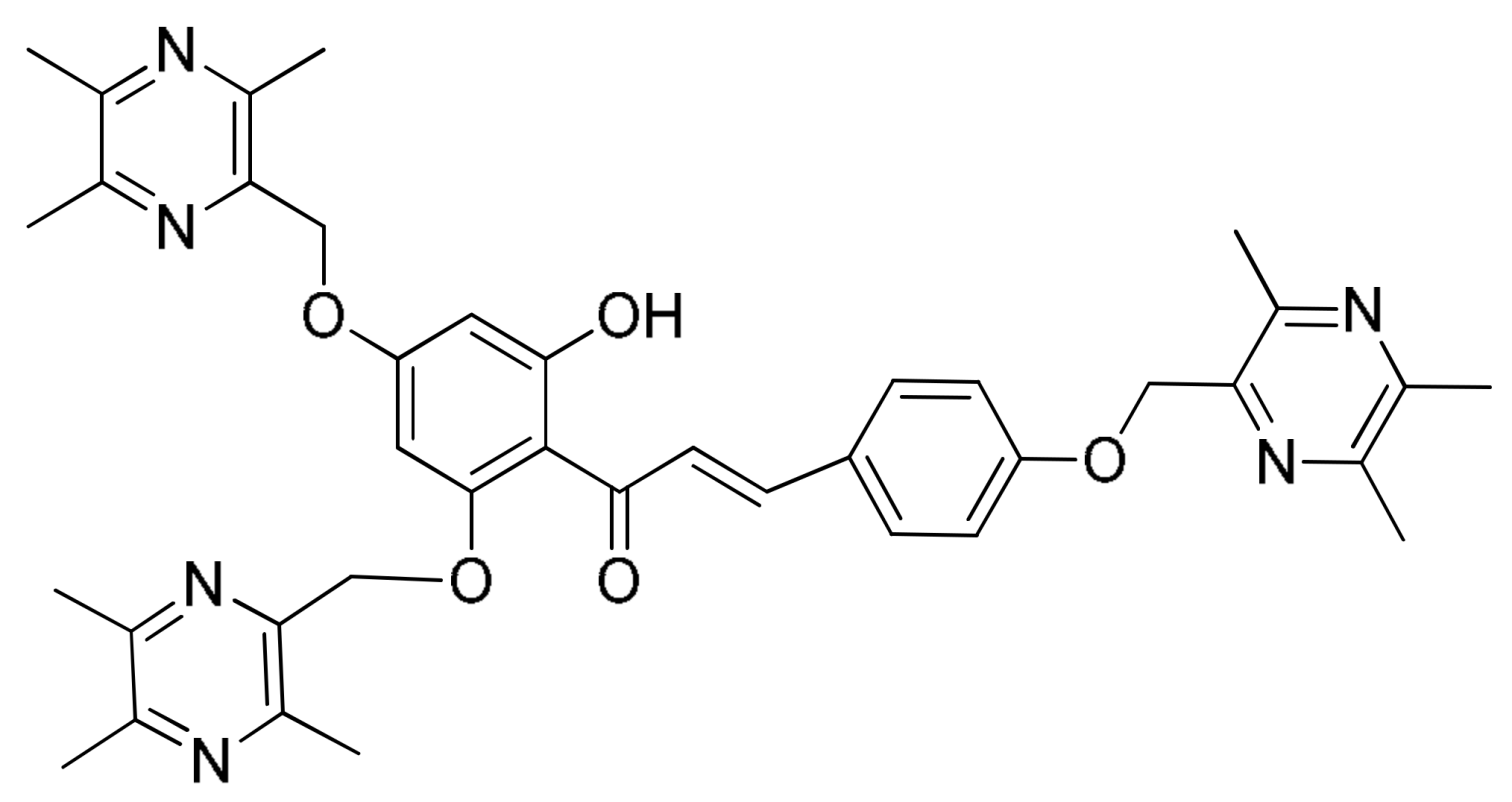	**15**	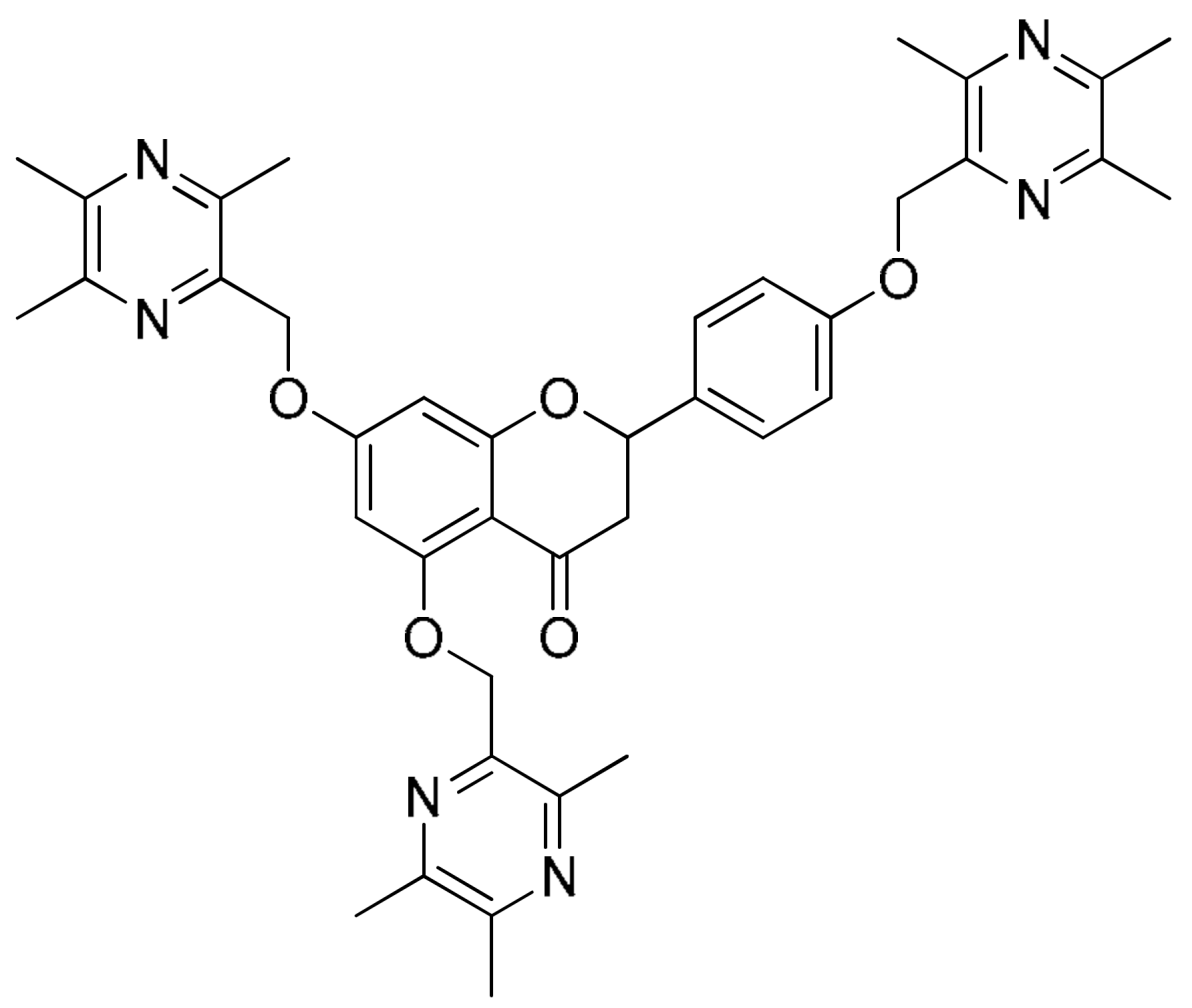
**16**	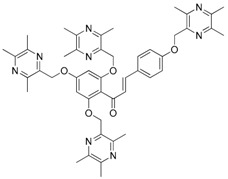	**17**	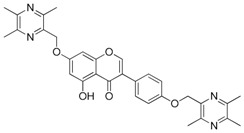
**18**	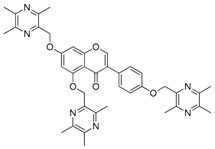	**19**	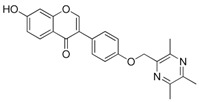
**20**	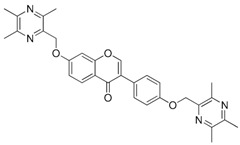	**21**	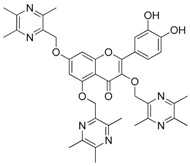
**22**	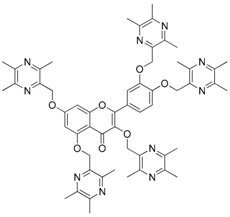	**23**	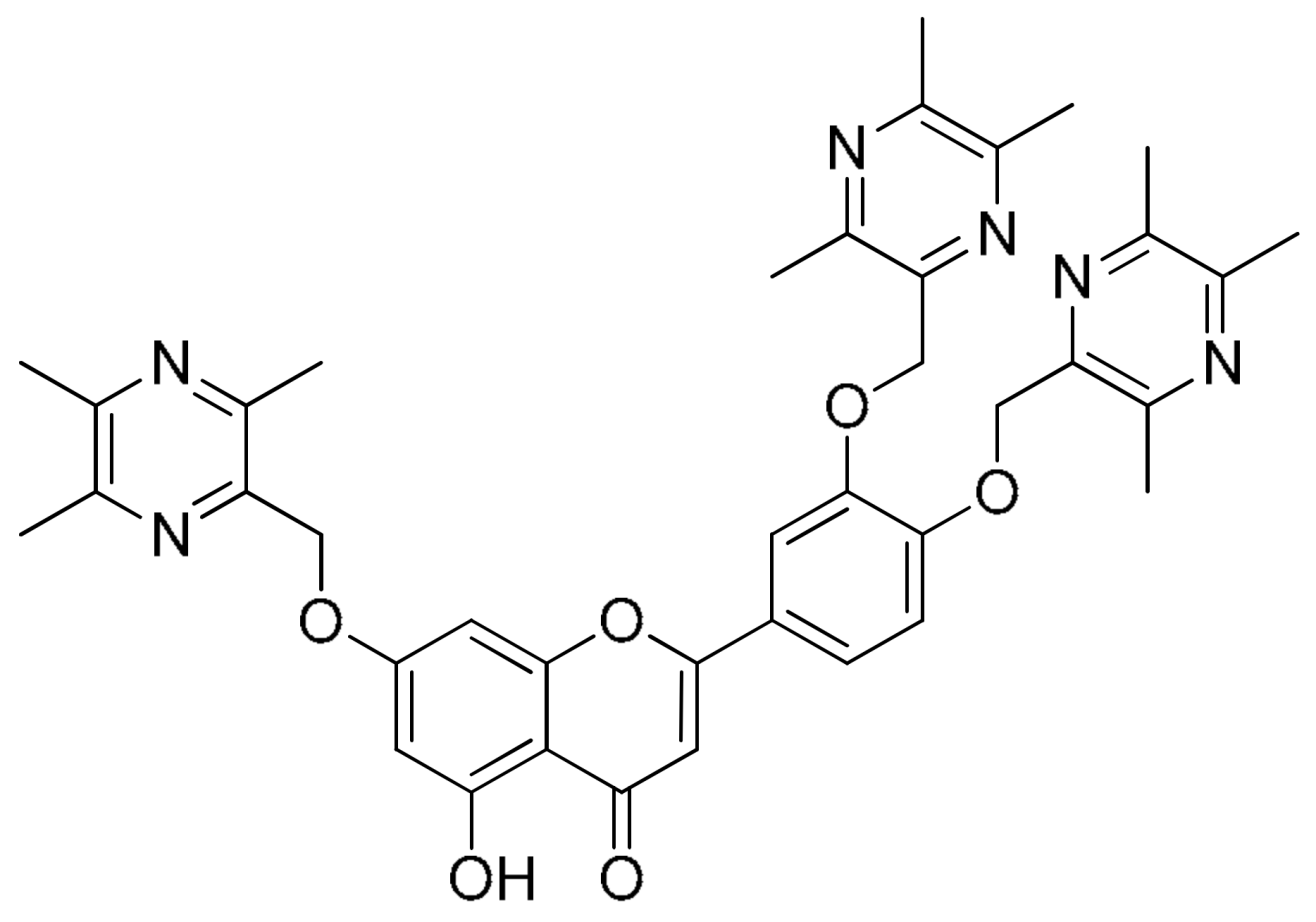
**24**	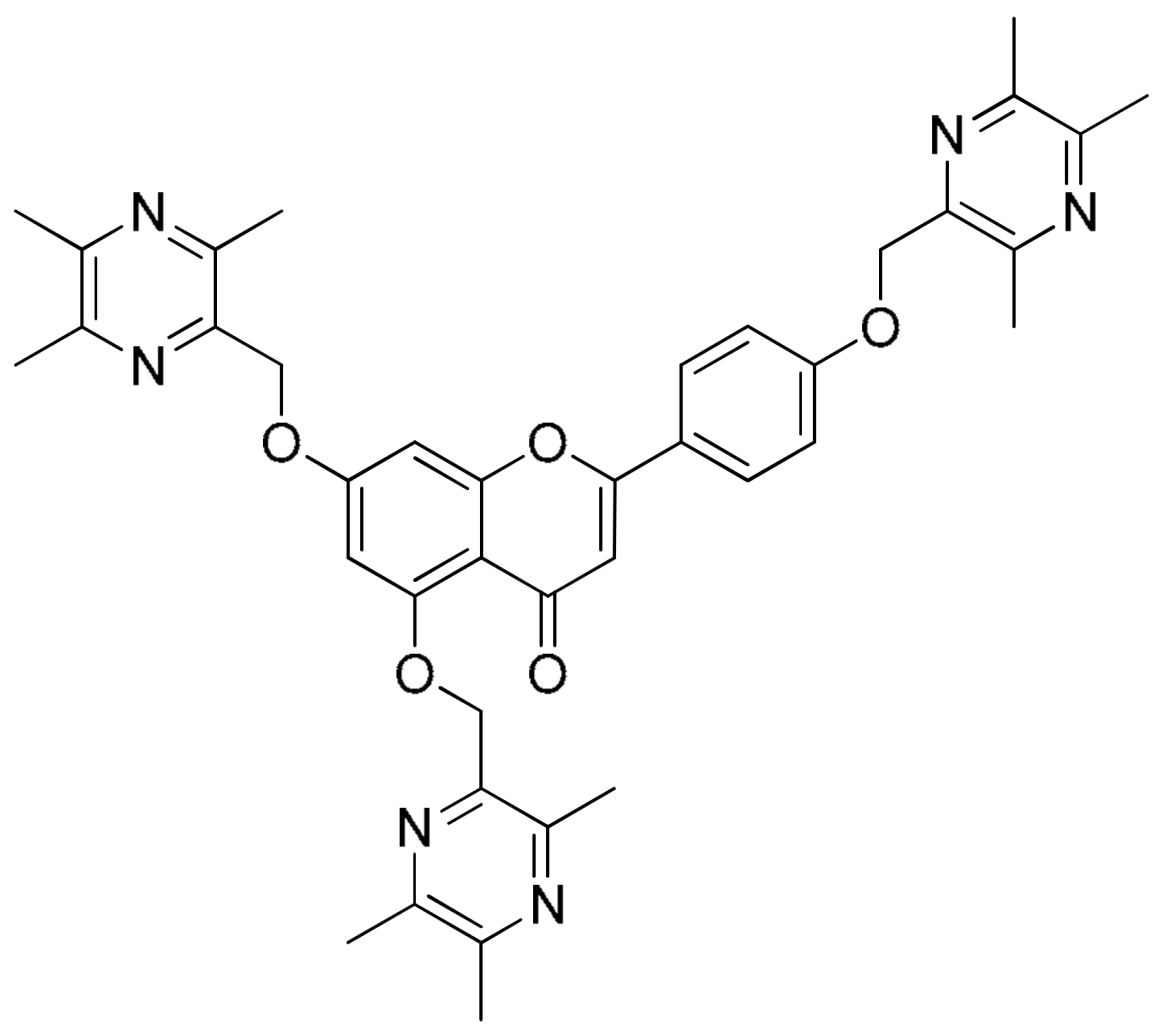	**25**	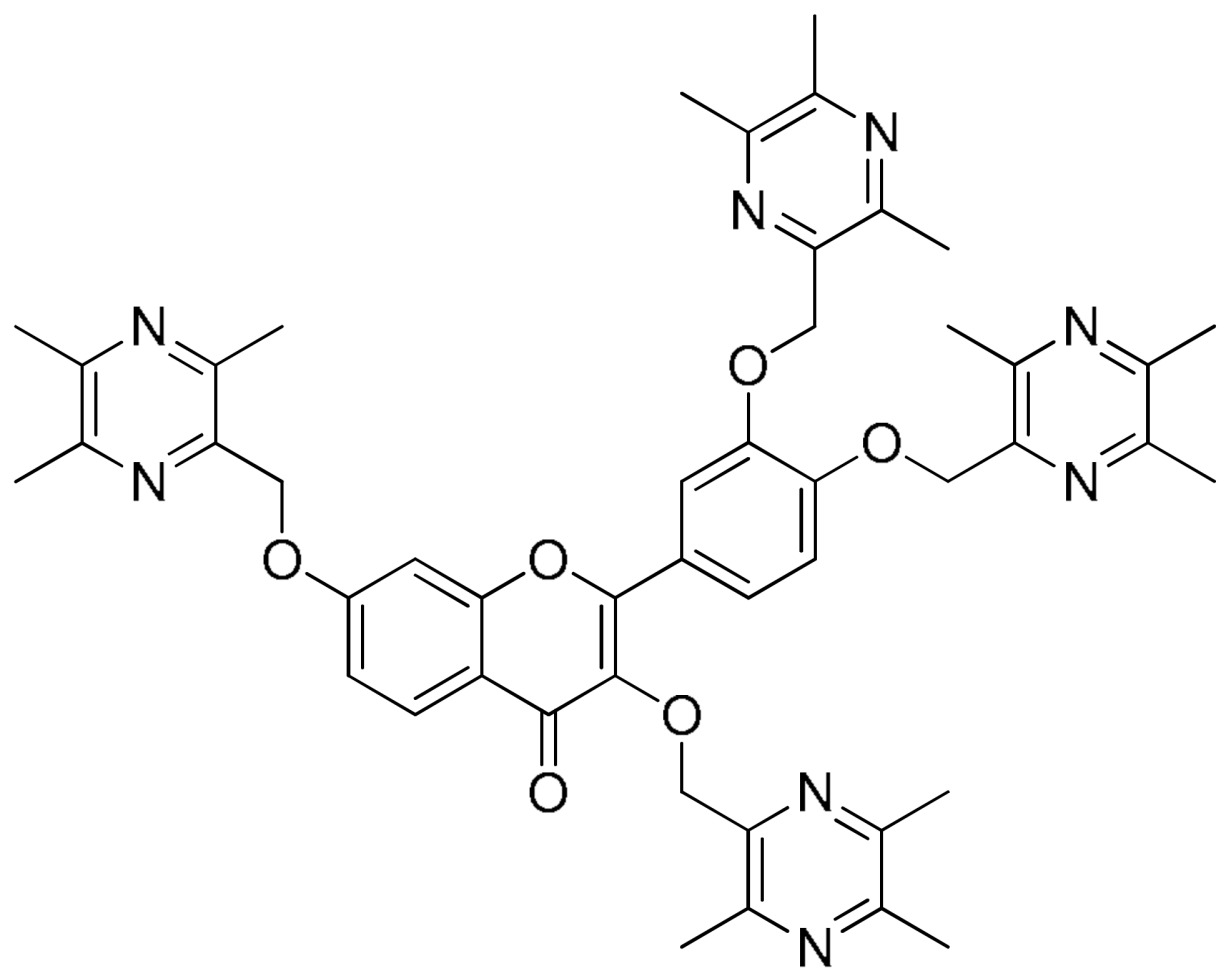

**Table 3:** IC_50_ values of TMP-flavonoid derivatives and raw materials on HepG-2, Bel-7402, HT-29, MCF-7 and HeLa for 72 h. Data were expressed as inhibitory ratio ± SD based on three independent experiments (*n* = 3).

Compound	IC_50_ (μM)				
	HepG-2	Bel-7402	HT-29	MCF-7	HeLa
**Luteolin**	11.83 ± 0.44	14.51 ± 0.68	19.24 ± 1.17	16.99 ± 0.31	21.28 ± 1.12
**Baicalein**	20.35 ± 2.07	21.59 ± 3.68	19.20 ± 2.72	37.88 ± 1.52	22.76 ± 3.54
**Apigenin**	16.11 ± 1.52	28.22 ± 0.38	23.44 ± 1.89	29.71 ± 1.43	30.58 ± 0.71
**Chrysin**	15.56 ± 0.30	35.00 ± 1.46	30.31 ± 1.96	32.06 ± 2.78	>40
**Quercetin**	13.57 ± 0.73	30.39 ± 2.51	27.00 ± 0.47	>40	20.83 ± 4.04
**Fisetin**	17.39 ± 1.04	25.46 ± 1.54	23.88 ± 0.81	38.10 ± 1.83	20.88 ± 1.65
**Genistein**	19.76 ± 1.31	34.66 ± 0.63	31.13 ± 0.26	35.62 ± 1.38	34.61 ± 2.52
**Daidzein**	21.81 ± 1.53	>40	>40	36.82 ± 1.48	>40
**10**	>40	>40	>40	>40	26.67±0.34
**13**	13.37 ± 2.50	26.13 ± 0.11	22.42 ± 0.35	>40	17.31 ± 1.07
**14**	17.31 ± 0.47	10.74 ± 1.12	31.88 ± 1.96	29.79 ± 2.18	25.11 ± 1.80
**16**	20.62 ± 1.39	>40	34.39 ± 2.32	16.54 ± 0.45	30.50 ± 2.62
**17**	>40	>40	10.67 ± 1.35	>40	>40
**19**	14.49 ± 0.48	28.87 ± 0.49	11.72 ± 1.29	10.43 ± 1.23	14.31 ± 1.17
**20**	>40	25.16 ± 1.72	10.90 ± 2.30	>40	16.48 ± 1.79
**DDP**	4.14 ± 0.16	6.73 ± 0.37	5.83 ± 0.59	6.75 ± 0.57	4.76 ± 0.41

IC_50_ values: when the IC_50_ values of the compounds were higher than 40 for all cells, the groups of data were not listed in the table.

**Table 4:** Proliferation rates of TMP-flavonoid derivatives on HUVEC-12 for 24 h. Data were expressed as inhibitory ratio ± SD based on three independent experiments (*n* = 3).

Compound	Proliferation Rate (%)					IC_50_ (μM)
	2.5 μM	5 μM	10 μM	20 μM	40 μM	
**14**	1.62 ± 0.75	1.99 ± 0.23	14.84 ± 1.41	16.71 ± 0.29	−2.32 ± 1.37	>40
**19**	−8.75 ± 0.38	−19.35 ± 0.35	−19.49 ± 2.17	−24.22 ± 3.19	−25.07 ± 1.02	>40
**DDP**	−23.93 ± 0.75	−31.38 ± 1.46	−58.24 ± 0.82	−66.58 ± 1.75	−82.69 ± 2.19	9.11 ± 0.54
